# Integrated mRNA, sRNA, and degradome sequencing reveal oilseed rape complex responses to *Sclerotinia sclerotiorum* (Lib.) infection

**DOI:** 10.1038/s41598-018-29365-y

**Published:** 2018-07-20

**Authors:** Hongju Jian, Jinqi Ma, Lijuan Wei, Pu Liu, Aoxiang Zhang, Bo Yang, Jiana Li, Xinfu Xu, Liezhao Liu

**Affiliations:** Chongqing Engineering Research Center for Rapeseed, College of Agronomy and Biotechnology, Southwest University, Beibei, Chongqing 400715 P. R. China

## Abstract

Sclerotinia stem rot (SSR), caused by the fungal pathogen *Sclerotinia sclerotiorum*, is a devastating disease resulting in yield losses and decreases in seed quality in oilseed rape (*Brassica napus*) worldwide. However, the molecular mechanisms underlying the response of oilseed rape to *S*. *sclerotiorum* infection at the transcriptional and post-transcriptional levels are poorly understood. Here, we used an integrated omics approach (transcriptome, sRNAome, and degradome sequencing) on the Illumina platform to compare the RNA expression and post-transcriptional profiles of oilseed rape plants inoculated or not with *S*. *sclerotiorum*. In total, 7,065 differentially expressed genes (DEGs) compared with the mock-inoculated control at 48 hours post inoculation were identified. These DEGs were associated with protein kinases, signal transduction, transcription factors, hormones, pathogenesis-related proteins, secondary metabolism, and transport. In the sRNA-Seq analysis, 77 known and 176 novel miRNAs were identified; however, only 10 known and 41 novel miRNAs were differentially expressed between the samples inoculated or not with *S*. *sclerotiorum*. Degradome sequencing predicted 80 cleavage sites with 64 miRNAs. Integrated mRNA, sRNA and degradome sequencing analysis reveal oilseed rape complex responses to *S*. *sclerotiorum* infection. This study provides a global view of miRNA and mRNA expression profiles in oilseed rape following *S*. *sclerotiorum* infection.

## Introduction

Oilseed rape (*Brassica napus* L.), one of the most economically important crops worldwide, is a valued source of vegetable oil, animal feed, and bioenergy^1^. Among the fungal diseases affecting oilseed rape, Sclerotinia stem rot, caused by *Sclerotinia sclerotiorum* (Lib.) de Bary, poses a significant threat to yield and seed quality. *S*. *sclerotiorum* is a necrotrophic pathogen, which causes severe disease in over 400 plant species, such as soybean (*Glycine max*), sunflower (*Hellianthus annuus*), and chickpea (*Cicer arietinum*)^2^. Infection occurs through senescing or injured plant tissues, lower stems, and flower petals^3^. Necrotic lesions develop quickly throughout the stem and leaves, resulting in wilting, necrosis, stem breakage, yield losses, and decreases in seed quality. *S*. *sclerotiorum* can persist in the soil for many years and it is not a good way to suppress the pathogen using fungicides and biocontrol agents. Breeding of resistant oilseed rape cultivars is the most viable option for controlling the disease; however, the molecular mechanisms underlying the interaction between *S*. *sclerotiorum* and *B*. *napus* are not clear.

Forward genetic approaches, such as quantitative trait loci (QTL) and genome-wide association studies (GWAS), have been used to map candidate genes for *S*. *sclerotiorum* resistance, and pathogen-resistant QTLs have been identified on almost every chromosome^4–11^. Genes encoding mitogen-activated protein kinases (MAPKs)^12^, WRKY transcription factors (TFs)^13,14^, and germin-like proteins^15^ and several plant hormones (e.g., jasmonic acid, salicylic acid, and ethylene)^16,17^ have been shown to play important roles in the resistance to *S*. *sclerotiorum*. RNA sequencing (RNA-Seq) is a powerful tool for characterizing the pathogen response pathways and genes involved in resistance in many crops, including *Oryza sativa* (rice)^18^, *Triticum aestivum* (wheat)^19^, *Sorghum bicolor* (sorghum)^20^, sugarcane^21^, and cucumber^22^. Transcriptome studies have shown that *B*. *napus* genes involved in the response to pathogen invasion (e.g., genes encoding receptor-like kinases, proteins harboring nucleotide binding site leucine-rich repeats, proteins that function in mitogen-activated protein kinase cascades, G-proteins, calcium binding proteins, hormones, TFs, pathogenesis-related (PR) proteins, proteins involved in phenylpropanoid and glucosinolate metabolism, and transport proteins) were significantly altered following infection^10,23,24^.

MicroRNAs (miRNAs) are endogenous, non-coding RNAs that play crucial roles in various biological processes at the transcriptional and post-transcriptional level by negatively regulating gene expression^25^. miRNAs act as key components in gene regulatory pathways associated with many processes, such as germination^25^, development^26^, organ maturation^27^, signal transduction^28^, and stress responses^29^. Moreover, several studies have shown that miRNAs function in responses to pathogen invasion^29,30^. miR393 contributed to the defense response in *Arabidopsis thaliana* against *Pseudomonas syringae* pv. *tomato* (Pst) by suppressing target genes, including auxin receptors, transport inhibitor response 1 (TIR1), auxin signaling F-box protein 2 (AFB2), and AFB3, which negatively regulate auxin signaling^29^. Furthermore, miR393 acts as a key regulator in the glucosinolate pathway, which is involved in plant responses to pathogens^31^. In *Brassica rapa*, miR158 and miR1885 were induced after infection by *Turnip mosaic virus* (TuMV) and suppressed disease resistance protein genes -Nucleotide-binding site leucine-rich repeat^32^. MiR396 from *A*. *thaliana* functions as a positive regulator during cyst nematode infection^33^. In *Nicotiana tabacum* (tobacco), miR6019 and miR6020 guided to incise N genes, which increases resistance to the *Tobacco mosaic virus* (TMV)^34^.

Several studies have been conducted to identify miRNAs in oilseed rape. Xie *et al*.^35^ first identified 21 potential *B*. *napus* miRNAs using computational methods. Later, next generation sequencing technologies were used to identify miRNAs in different tissues or in response to different stresses^36–41^. There are only 92 mature miRNA sequences of *B*. *napus* in the miRBase database (version: 21.0). Compared with the number of miRNAs that have been identified in other species, such as *A*. *thaliana*, *Arabidopsis lyrata*, and *Oryza sativa*. It is likely that there are many *B. napus* miRNAs that have not yet been discovered.

The molecular mechanisms underlying the interaction between *S*. *sclerotiorum* and *B*. *napus* are only partially understood, due to the lack of resistant lines. We identified five relatively resistant lines in a previous study^10^. To investigate the response of oilseed rape to *S*. *sclerotiorum* infection at the mRNA and miRNA levels, we performed RNA-Seq, sRNA-Seq, and degradome deep sequencing on both mock-inoculated and *S*. *sclerotiorum* inoculated stems at 48 hours post-inoculation (hpi). We identified differentially expressed genes associated with protein kinases, signal transduction (CDPKs, G proteins, and MAPKs), TFs, hormones, PR proteins, secondary metabolism, and transport proteins. In sRNA-Seq analysis, 10 known and 41 novel miRNAs were differentially expressed at 48 hpi with *S*. *sclerotiorum*. Furthermore, 80 cleavage sites with 64 miRNAs were predicted by degradome sequencing. Our data provide a broad view of gene expression changes after*S*. *sclerotiorum* infection in oilseed rape.

## Results

### Overview of transcriptome sequencing and analysis of gene expression

To identify the expression levels of *B*. *napus* genes, cDNA libraries were constructed from the stems of the inoculated plants (48 hours after inoculation with *S*. *sclerotiorum* (T48)) and the mock-inoculated control (CK) and paired-end (PE150) sequenced by HiSeqTM 2500. After filtering low quality reads and removing sequences with N, 44,817,708 reads from the T48 and 42,143,988 reads from the CK libraries were obtained. Furthermore, 67.37% of the reads (30,192,484 reads) from the T48 library and 70.26% of the reads (29,611,119 reads) from the CK library were uniquely mapped to the *B*. *napus* genome (Table 1).

**Table 1:** Summary of Illumina transcriptome sequencing data mapping on *B*. *napus* reference genome for CK and T48 libraries.

Sample	Total Reads	mapped Reads	Uniq Map	Multiple Map	Pair Map	Single Map	Only Map Plus Strand	Only Map Minus Strand
T48	44817708	32180724	30192484	1988240	27463572	4717152	15775761	15779373
	100%	71.80%	67.37%	4.44%	61.28%	10.53%	35.20%	35.21%
CK	42143988	31191889	29611119	1580770	27143836	4048053	15276040	15303754
	100%	74.01%	70.26%	3.75%	64.41%	9.61%	36.25%	36.31%

**Figure 1:**
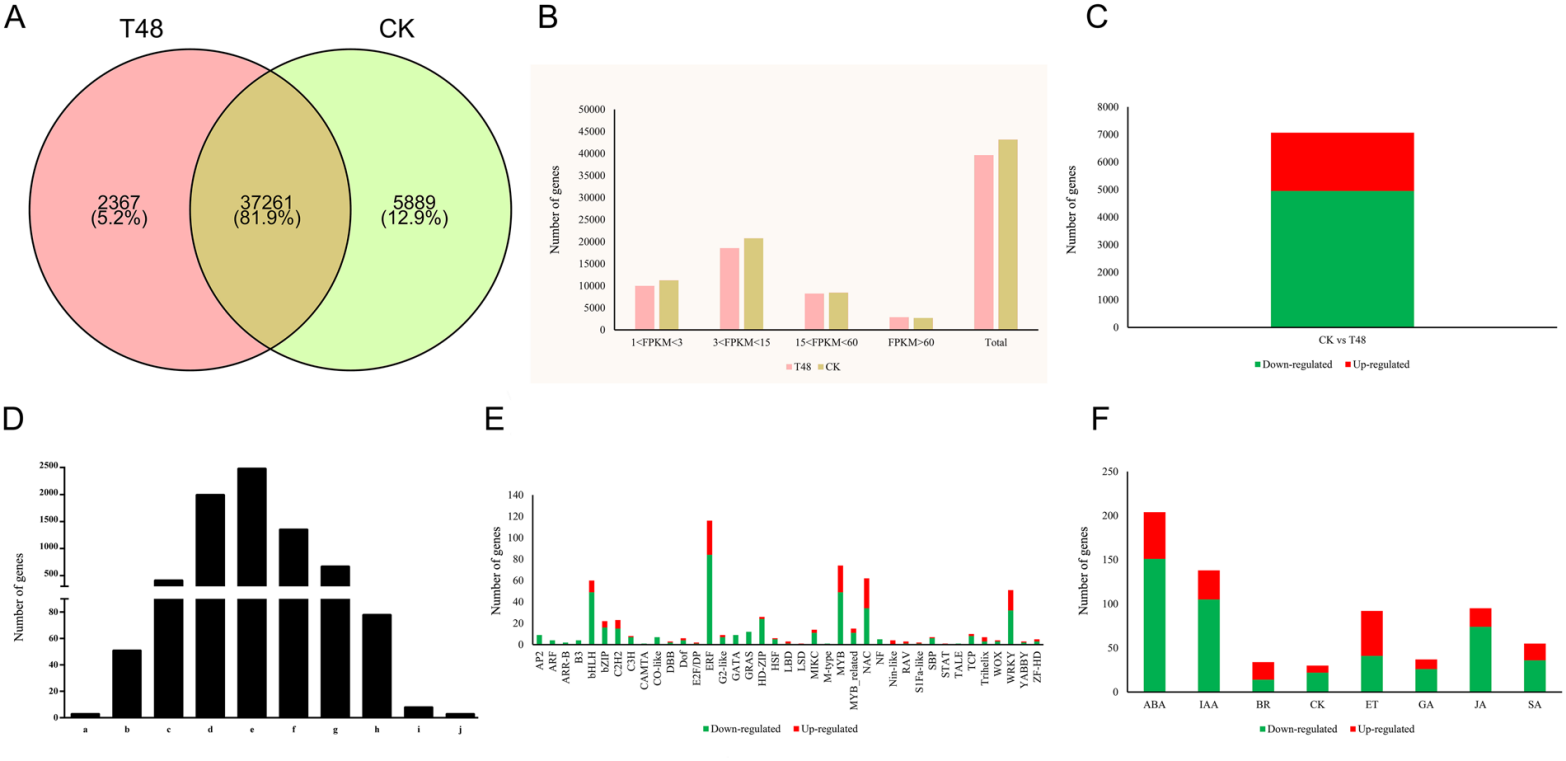
Gene expression profiles in two samples and DEGs identified at 48 hpi after *S*. *sclerotiorum* infection. (**A**) Venn Diagram of genes detected in the two samples. (**B**) Statistical analysis of gene expressions identified in two samples; (**C**) Statistical analysis of DEGs identified at 48 hpi after *S*. *sclerotiorum* infection; (**D**) Fold changes in the DEGs identified at 48 hpi after *S*. *sclerotiorum* infection. [(a) Number of DEGs with a log_2_ fold change ≤−9; (b) number of DEGs with −9 < log_2_ fold change ≤−7; (c) number of DEGs with −7 < log_2_ fold change ≤−5; (d) number of DEGs with −5 < log_2_ fold change ≤−3; (e) number of DEGs with −3 < log_2_ fold change ≤−1; (f) number of DEGs with 1 < log_2_ fold change ≤3; (g) number of DEGs with 3 <log_2_ fold change ≤5; (h) number of DEGs with 5 < log_2_ fold change ≤7; (i) number of DEGs with 7 < log_2_ fold change ≤9; (j) number of DEGs with log_2_ fold change ≥9]; (**E**) Statistical analysis of genes encoding TFs identified in the DEGs; (**F**) Statistical analysis of genes encoding hormones identified in the DEGs. [ABA: abscisic acid; IAA: auxin; BR: brassinosteroids; CK: cytokinin; GA: gibberellin JA: jasmonic acid; SA: Salicylic acid].

In total, 39,628 and 43,150 genes were detected in the T48 and CK libraries, respectively, using a FPKM (fragments per kilobase of transcript per million fragments mapped) value ≥1 with 37,261 genes in common between the two libraries, and 2,367 and 5,889 unique genes detected in the T48 and CK libraries, respectively (Fig. 1A). Moreover, almost all of the genes in the libraries showed moderate expression levels with only a small percentage of the genes expressed in high levels (Fig. 1B).

### Transcriptomic changes in response to *S*. *sclerotiorum* infection

In total, 7,065 genes were identified as DEGs with 4,950 down-regulated and 2,115 up-regulated genes between the inoculated and mock-inoculated samples with a cut-off FDR (false discovery rate) value of <0.001 and |log_2_ (fold change) | ≥1 (Fig. 1C). As shown in Fig. 1D, more than 92% of the DEGs had a fold change ≤32 and only a few DEGs had a fold change ≥128.

Transcription factors (TFs) play important roles in plant biotic stress responses^14^. To further analyze the function of the DEGs, we aligned all DEGs against known TFs in the *A*. *thaliana* genome. In total, 602 DEGs belonging to 39 TF families were identified with 434 being down-regulated and 168 being up-regulated and the |log2 (fold change)| of most TF genes were between 1 and 5 (Fig. 1E). The TF family members were unevenly distributed; five TF families, *ERF* (116), *MYB* (74), *NAC* (62), *bHLH* (60), and *WRKY* (51) accounted for more than 60% (363/602) of all TFs. Furthermore, most members of *ERF* (84/116), *MYB* (49/74), *WRKY* (32/51), *bHLH* (49/60), *bZIP* (16/22), and *HD-ZIP* (24/26) were down-regulated. Plant hormones, such ethylene (ET), jasmonic acid (JA), and salicylic acid (SA), play vital roles in biotic stresses. In total, 685 DEGs belonging to eight classifications of plant hormones were detected with 469 being down-regulated and 216 being up-regulated. Almost all of the ABA (151/204), IAA (105/138), JA (74/95), SA (36/55), GA (26/37), and CK (22/30) genes were down-regulated and the ET and BR genes were up- and down-regulated to similar extents (Fig. 1F).

### Functional classifications of the DEGs

**Figure 2:**
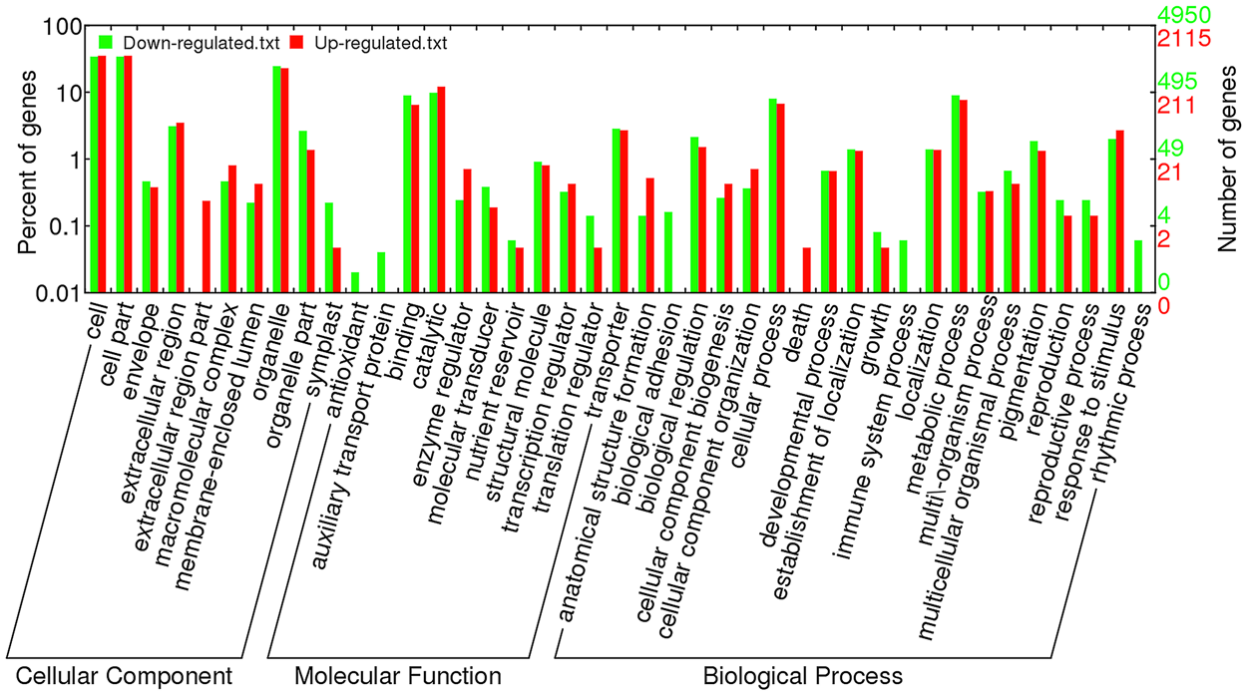
Gene Ontology (GO) classification of DEGs identified at 48 hpi after *S*. *sclerotiorum* infection.

**Figure 3:**
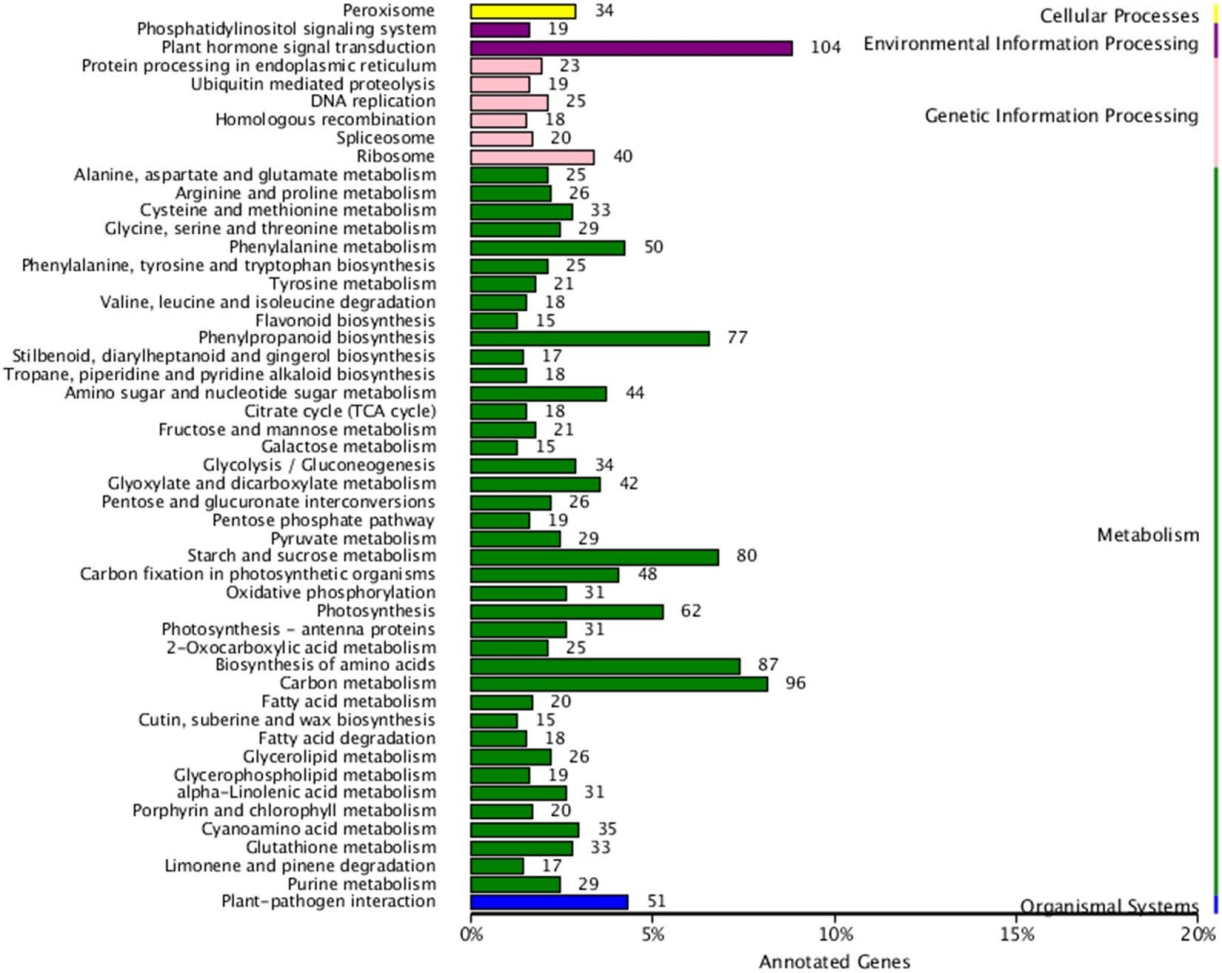
KEGG pathways of DEGs identified at 48 hpi after *S*. *sclerotiorum* infection.

To further explore the functions of the DEGs in response to *S*. *sclerotiorum* infection, Gene Ontology (GO) enrichment analysis was conducted on the down- and up-regulated DEGs. We found 10, 11, and 20 functional groups in cellular components (CC), molecular functions (MF), and biological processes (BP), respectively (Fig. 2). Moreover, the dominant functions in each of the three main categories were cellular processes (GO: 0009987) and metabolic processes (GO: 0008152) in the BP category, cell (GO: 0005623), cell part (GO: 0044464), and organelle (GO: 0043226) in the CC category, and binding (GO: 0005488) and catalytic activity (GO: 0008152) in the MF category (Fig. 2). In addition, the DEGs were aligned against the KEGG pathways database to identify pathways that were responsive to *S*. *sclerotiorum* infection. In total, 50 pathways were detected in our study including five branches: cellular processes, environmental information processing, genetic information processing, metabolism, and organismal systems. The most abundant pathway in each branch was peroxisome (34), plant hormone signal transduction (104), ribosome (40), carbon metabolism (96), and plant-pathogen interaction (51), respectively (Fig. 3 and Table S1).

**Figure 4:**
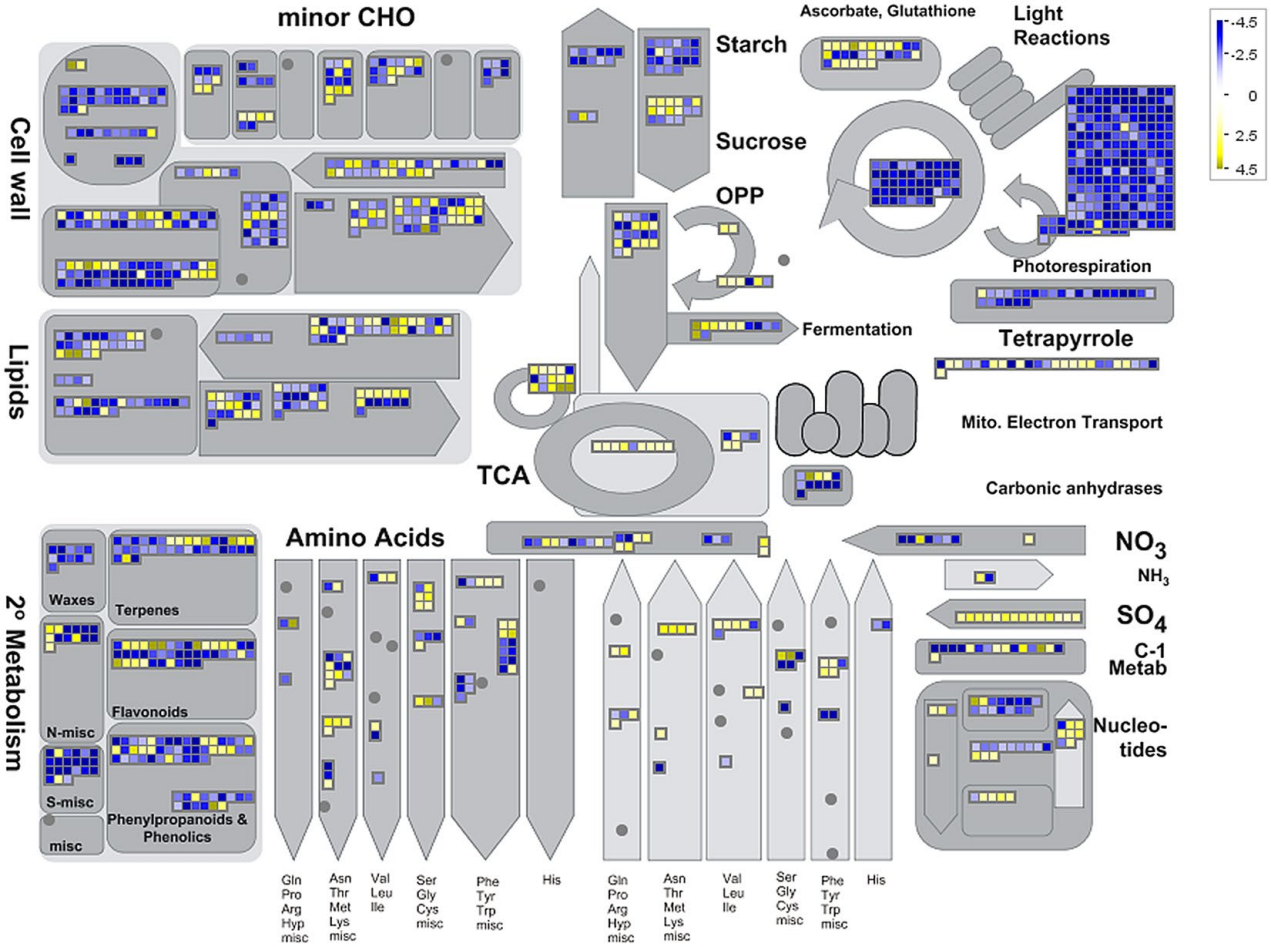
Overview of DEGs involved in different metabolic processes at 48 hpi after *S*. *sclerotiorum* infection. The images were drew using MapMan and show different functional categories of all DEGs identified in this study. Each box depicts an individual gene. Yellow and blue were represented up-DEGs and down-DEGs, respectively. The scale bar represents fold change values.

**Figure 5:**
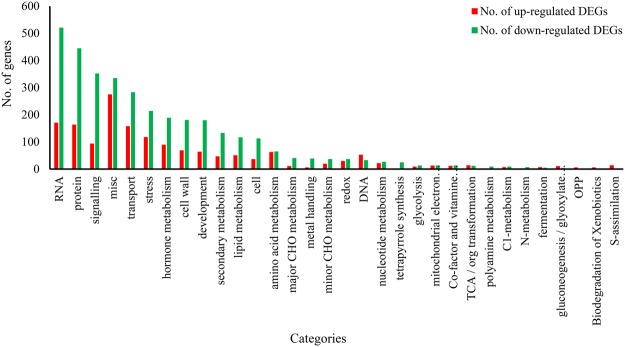
Overview of the total number of DEGs distribution in the changed pathways using the MapMan. The number of up-regulated and down-regulated genes within each category is represented by red and green bars, respectively. See S3 Tables for the detailed information of the changed pathways.

To decipher the mechanisms of the resistance response to *S*. *sclerotiorum* infection in *B*. *napus*, MapMan software was used to classify and determine the metabolic pathways of the DEGs. As shown in Fig. 4, photosynthesis, glyoxalic acid, and carbon metabolism were suppressed, while cell wall, lipids, and metabolism (sulphur metabolism and amino acid metabolism) were mostly activated. An overview of the DEGs represented in the different processes is provided in Fig. 5 and Table S2.

### Validation of RNA-Seq results by qRT-PCR

**Figure 6:**
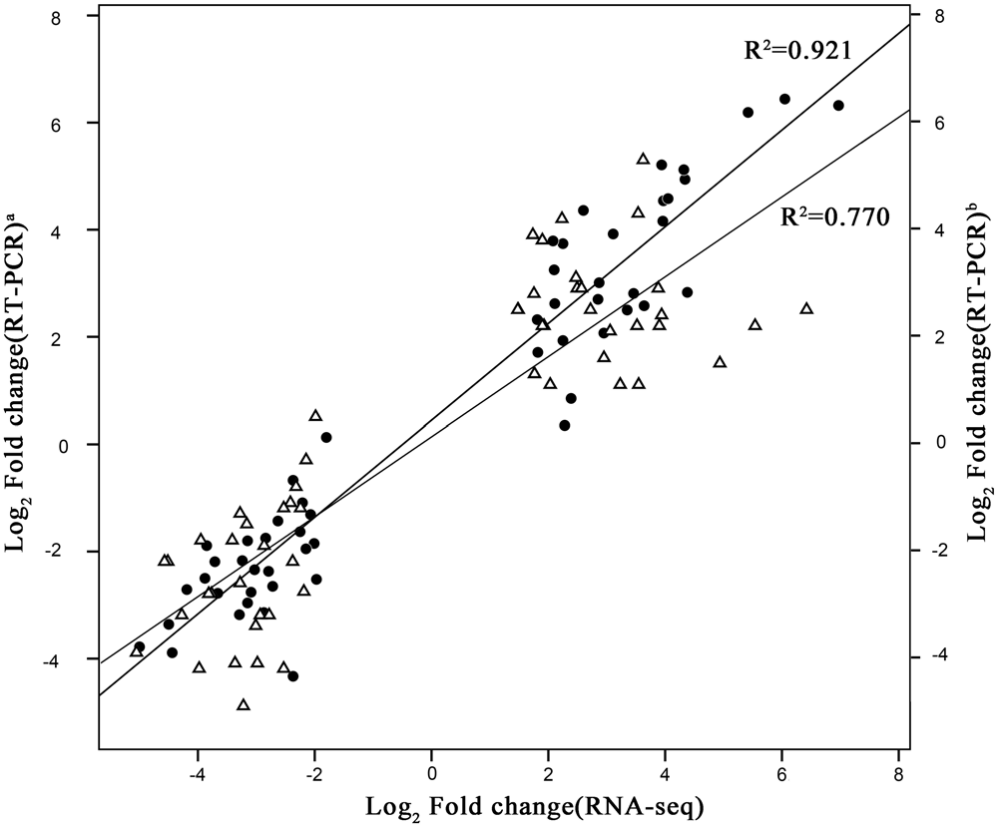
Correlation of gene expression between qPCR and RNA-seq approaches. The qPCR log2 value of the expression ratio (inoculated/mock-inoculated) (y-axis) was plotted against the value from the RNA-seq (x-axis). a, data from resistant lines; b, data from ZS11. All qPCR data were collected from three biological replicates and three technical replicates for each sample.

qRT-PCR was used to validate the data from RNA-Seq. In total, 55 genes (hormones, TFs, MAPKs, NBS-LRRs, MLO, and lignin synthesis) were selected for the qPCR assays (Table S3). High correlations were obtained between qPCR and RNA-Seq techniques. The correlation coefficient between qPCR and RNA-Seq data obtained from resistant lines or ZS11 were 0.921 and 0.770 (Fig. 6), emphasizing the reproducibility and reliability of the RNA-Seq data.

### sRNA library construction

**Figure 7:**
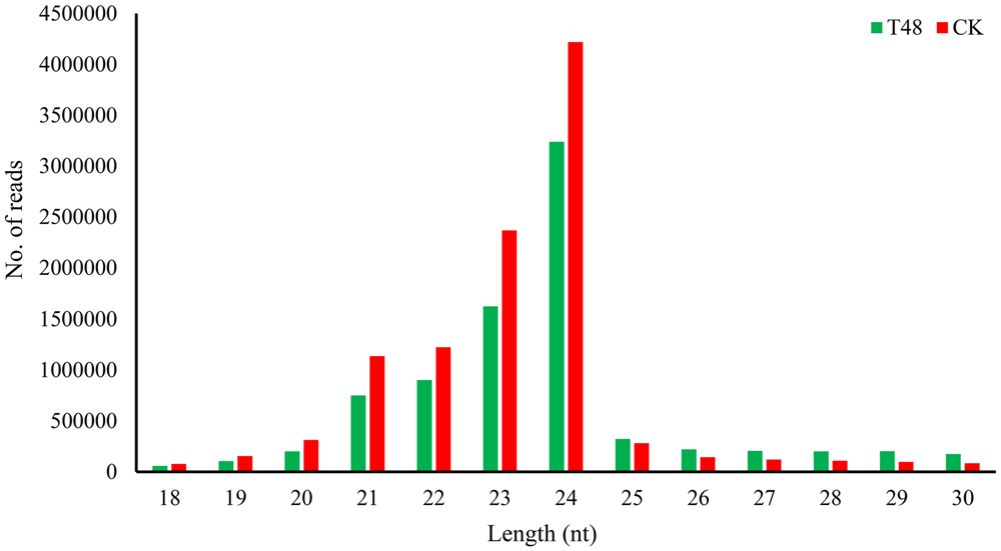
Length distribution of sRNAs detected in two libraries.

To elucidate the regulatory roles of the miRNAs responsive to *S*. *sclerotiorum* infection, two sRNA libraries were constructed using total RNA isolated from the inoculated and mock-inoculated samples. After sequencing, a total of 42,806,114 and 32,205,058 high-quality, raw sequence reads were obtained from the T48 and CK libraries, respectively. After removing adapters, poly-A sequences, and short RNA reads smaller than 18 nucleotides (nt), 26,570,562 and 23,051,268 clean reads were obtained from the T48 and CK libraries, respectively. After further filtering the RFam and Repbase sequences, 15,459,745 (58.18%) and 17,767,719 (77.08%) small RNA sequences from the T48 and CK libraries, respectively, were mapped to the *B*. *napus* genome using SOAP2 (Table 2). Similar patterns of length distribution of the sRNAs between the two libraries are shown in Fig. 7, which revealed that the majority of the reads were 21–24 nt in length, and 24-nt reads were the most abundant (Fig. 7). Furthermore, the common and specific of total and unique small RNA sequences were compared between the two libraries), showing that 63.87% and 10.07% of the total sRNAs and unique sRNA were shared, respectively.

**Table 2:** Distribution of small RNAs among different categories.

Types	T48		CK	
	Number	Percentage	Number	Percentage
Total	26570562	100.00%	23051268	100.00%
rRNA	8767858	33.00%	4296731	18.64%
scRNA	0	0.00%	0	0.00%
snRNA	0	0.00%	0	0.00%
snoRNA	21392	0.08%	27068	0.12%
tRNA	2231675	8.40%	844134	3.66%
Repbase	89892	0.34%	115616	0.50%
Unannotated	15459745	58.18%	17767719	77.08%

### Identification of known and novel miRNAs in *B*. *napus*

**Figure 8:**
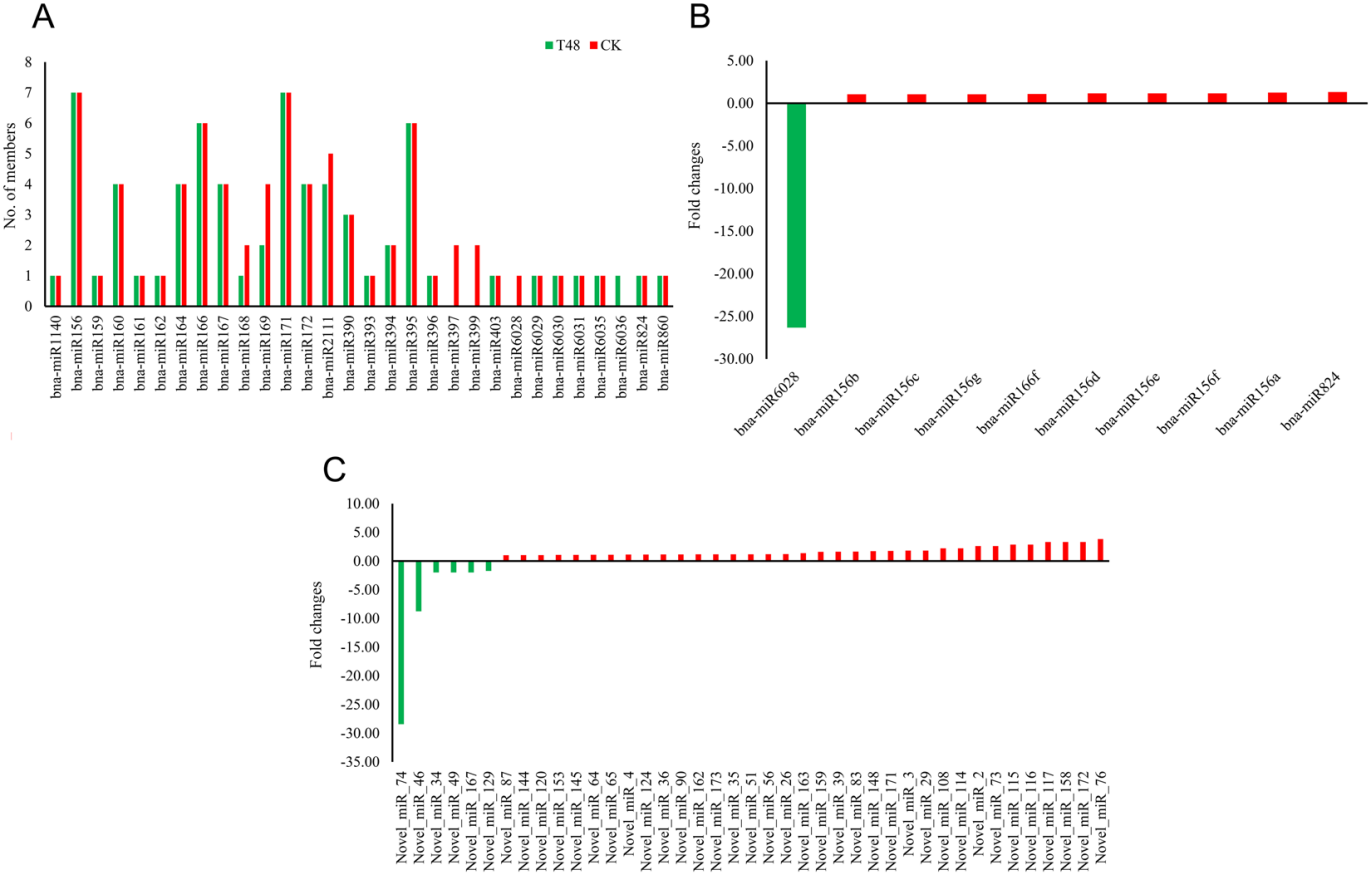
The number of known miRNAs members and differentially expressed miRNAs between CK and T48 libraries. (**A**) Number of family members per miRNA family detected in the two libraries. (**B**) expressed differentially of known miRNAs; (**C**) expressed differentially of novel miRNAs.

To identify known miRNAs in the two libraries, sRNA sequences obtained from each library were aligned with mature sequences of *B*. *napus* miRNAs deposited in miRBase v21.0. A total of 77 known miRNAs belonging to 30 families were identified in the T48 (68 miRNAs) and CK (76 miRNAs) libraries (Table 3); among them, 67 (87.01%) were detected in both libraries. Among the 30 families, bna-miR156 and bna-miR171 had seven members in both libraries, comprising the largest groups, followed by bna-miR166 and bna-miR395 with six members (Fig. 8A). In our study, 33.82% (23 miRNAs) and 38.16% (29 miRNAs) of the miRNAs had more than 100 reads in the T48 and CK libraries, respectively, and 16.18% (11 miRNAs) and 17.11% (13 miRNAs) of the miRNAs had more than 1,000 reads in the T48 and CK libraries, respectively. Among them, bna-miR159 had the highest expression level in both libraries (48,352 and 71,828 reads in the T48 and CK libraries, respectively, Table 3).

**Table 3:** The information of known miRNAs identified in CK and T48 libraries.

Name of miRNAs	Read Counts		Length	Expression Normalization		Mature Sequence
	T48	CK		T48	CK	
bna-miR1140	3	7	21	2.69	3.46	ACAGCCTAAACCAATCGGAGC
bna-miR156a	25	19	21	22.41	9.40	TGACAGAAGAGAGTGAGCACA
bna-miR156b	55	48	21	49.29	23.74	TTGACAGAAGATAGAGAGCAC
bna-miR156c	55	48	21	49.29	23.74	TTGACAGAAGATAGAGAGCAC
bna-miR156d	21	17	20	18.82	8.41	TGACAGAAGAGAGTGAGCAC
bna-miR156e	21	17	20	18.82	8.41	TGACAGAAGAGAGTGAGCAC
bna-miR156f	21	17	20	18.82	8.41	TGACAGAAGAGAGTGAGCAC
bna-miR156g	55	48	21	49.29	23.74	TTGACAGAAGATAGAGAGCAC
bna-miR159	48352	71828	21	43334.40	35518.82	TTTGGATTGAAGGGAGCTCTA
bna-miR160a	42	116	21	37.64	57.36	TGCCTGGCTCCCTGTATGCCA
bna-miR160b	42	116	21	37.64	57.36	TGCCTGGCTCCCTGTATGCCA
bna-miR160c	42	116	21	37.64	57.36	TGCCTGGCTCCCTGTATGCCA
bna-miR160d	42	116	21	37.64	57.36	TGCCTGGCTCCCTGTATGCCA
bna-miR161	7	10	21	6.27	4.94	TCAATGCACTGAAAGTGACTA
bna-miR162a	1	1	21	0.90	0.49	TCGATAAACCTGTGCATCCAG
bna-miR164a	1075	3252	21	963.44	1608.11	TGGAGAAGCAGGGCACGTGCA
bna-miR164b	235	744	21	210.61	367.91	TGGAGAAGCAGGGCACGTGCG
bna-miR164c	235	744	21	210.61	367.91	TGGAGAAGCAGGGCACGTGCG
bna-miR164d	235	744	21	210.61	367.91	TGGAGAAGCAGGGCACGTGCG
bna-miR166a	5126	10238	21	4594.06	5062.67	TCGGACCAGGCTTCATTCCCC
bna-miR166b	5126	10238	21	4594.06	5062.67	TCGGACCAGGCTTCATTCCCC
bna-miR166c	5126	10238	21	4594.06	5062.67	TCGGACCAGGCTTCATTCCCC
bna-miR166d	5126	10238	21	4594.06	5062.67	TCGGACCAGGCTTCATTCCCC
bna-miR166e	5126	10238	21	4594.06	5062.67	TCGGACCAGGCTTCATTCCCC
bna-miR166f	94	80	21	84.25	39.56	TCGGACCAGGCTTCATCCCCC
bna-miR167a	44	89	22	39.43	44.01	TGAAGCTGCCAGCATGATCTAA
bna-miR167b	44	89	22	39.43	44.01	TGAAGCTGCCAGCATGATCTAA
bna-miR167c	43	89	21	38.54	44.01	TGAAGCTGCCAGCATGATCTA
bna-miR167d	30	77	20	26.89	38.08	TGAAGCTGCCAGCATGATCT
bna-miR168a	1088	1681	21	975.10	831.25	TCGCTTGGTGCAGGTCGGGAA
bna-miR168b	0	3	21	0.00	1.48	TCGCTTGGTGCAGGTCGAGAA
bna-miR169a	0	1	21	0.00	0.49	CAGCCAAGGATGACTTGCCGA
bna-miR169b	0	1	21	0.00	0.49	CAGCCAAGGATGACTTGCCGA
bna-miR169m	24	83	21	21.51	41.04	TGAGCCAAAGATGACTTGCCG
bna-miR169n	10	6	21	8.96	2.97	CAGCCAAGGATGACTTGCCGG
bna-miR171a	3	17	21	2.69	8.41	TTGAGCCGTGCCAATATCACG
bna-miR171b	3	17	21	2.69	8.41	TTGAGCCGTGCCAATATCACG
bna-miR171c	3	17	21	2.69	8.41	TTGAGCCGTGCCAATATCACG
bna-miR171d	3	17	21	2.69	8.41	TTGAGCCGTGCCAATATCACG
bna-miR171e	3	17	21	2.69	8.41	TTGAGCCGTGCCAATATCACG
bna-miR171f	5	4	21	4.48	1.98	TGATTGAGCCGCGCCAATATC
bna-miR171g	5	4	22	4.48	1.98	TGATTGAGCCGCGCCAATATCT
bna-miR172a	1024	2651	21	917.74	1310.91	AGAATCTTGATGATGCTGCAT
bna-miR172b	78	204	21	69.91	100.88	GGAATCTTGATGATGCTGCAT
bna-miR172c	78	204	21	69.91	100.88	GGAATCTTGATGATGCTGCAT
bna-miR172d	290	836	21	259.91	413.40	AGAATCTTGATGATGCTGCAG
bna-miR2111a-3p	0	1	21	0.00	0.49	GTCCTCGGGATGCGGATTACC
bna-miR2111a-5p	3	3	21	2.69	1.48	TAATCTGCATCCTGAGGTTTA
bna-miR2111b-3p	2	9	21	1.79	4.45	ATCCTCGGGATACAGATTACC
bna-miR2111b-5p	3	3	21	2.69	1.48	TAATCTGCATCCTGAGGTTTA
bna-miR2111d	3	3	21	2.69	1.48	TAATCTGCATCCTGAGGTTTA
bna-miR390a	202	328	21	181.04	162.20	AAGCTCAGGAGGGATAGCGCC
bna-miR390b	202	328	21	181.04	162.20	AAGCTCAGGAGGGATAGCGCC
bna-miR390c	202	328	21	181.04	162.20	AAGCTCAGGAGGGATAGCGCC
bna-miR393	1	6	21	0.90	2.97	TCCAAAGGGATCGCATTGATC
bna-miR394a	935	1948	20	837.97	963.28	TTGGCATTCTGTCCACCTCC
bna-miR394b	935	1948	20	837.97	963.28	TTGGCATTCTGTCCACCTCC
bna-miR395a	4	17	21	3.58	8.41	CTGAAGTGTTTGGGGGAACTC
bna-miR395b	4	17	21	3.58	8.41	CTGAAGTGTTTGGGGGAACTC
bna-miR395c	4	17	21	3.58	8.41	CTGAAGTGTTTGGGGGAACTC
bna-miR395d	5	9	21	4.48	4.45	CTGAAGTGTTTGGGGGGACTC
bna-miR395e	5	9	21	4.48	4.45	CTGAAGTGTTTGGGGGGACTC
bna-miR395f	5	9	21	4.48	4.45	CTGAAGTGTTTGGGGGGACTC
bna-miR396a	1193	2099	21	1069.20	1037.95	TTCCACAGCTTTCTTGAACTT
bna-miR397a	0	5	22	0.00	2.47	TCATTGAGTGCAGCGTTGATGT
bna-miR397b	0	5	22	0.00	2.47	TCATTGAGTGCAGCGTTGATGT
bna-miR399a	0	1	21	0.00	0.49	TGCCAAAGGAGATTTGCCCGG
bna-miR399b	0	1	21	0.00	0.49	TGCCAAAGGAGATTTGCCCGG
bna-miR403	3727	5914	21	3340.24	2924.46	TTAGATTCACGCACAAACTCG
bna-miR6028	0	17	21	0.00	8.41	TGGAGAGTAAGGACATTCAGA
bna-miR6029	295	396	21	264.39	195.82	TGGGGTTGTGATTTCAGGCTT
bna-miR6030	145	159	22	129.95	78.63	TCCACCCATACCATACAGACCC
bna-miR6031	27	61	24	24.20	30.16	AAGAGGTTCGGAGCGGTTTGAAGC
bna-miR6035	3	4	21	2.69	1.98	TGGAGTAGAAAATGCAGTCGT
bna-miR6036	1	0	24	0.90	0.00	ATAGTACTAGTACTTGCATGATCA
bna-miR824	270	196	21	241.98	96.92	TAGACCATTTGTGAGAAGGGA
bna-miR860	7	2	21	6.27	0.99	TCAATACATTGGACTACATAT

**Figure 9:**
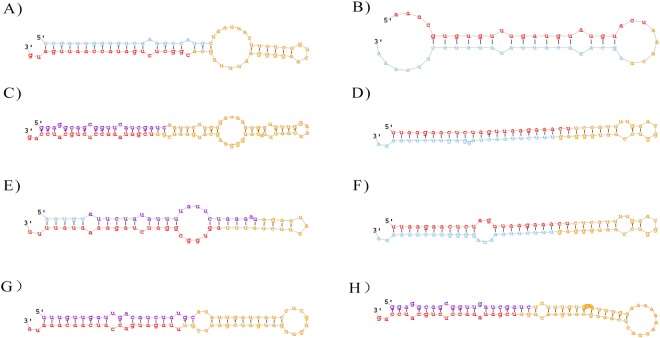
Predicted secondary structures of novel miRNAs in our study. The mature miRNAs are in red. (**A**) Novel_miR_2; (**B**) Novel_miR_4; (**C**) Novel_miR_11; (**D**) Novel_miR_22; (**E**) Novel_miR_119; (**F**) Novel_miR_133; (**G**) Novel_miR_137; (**H**) Novel_miR_147.

To predict novel miRNAs in *B*. *napus* from the two libraries, “unannotated” reads were aligned with the *B*. *napus* genome. A total of 176 novel miRNA candidates were obtained from the T48 (175 miRNAs) and CK (176 miRNAs) libraries; among them, Novel_miR_74 was expressed only in the CK library. The mature miRNAs were 20–25 nt in length with 21-nt reads being the most abundant (Table S4). The minimal folding energy (MFE) of these predicted pre-miRNAs ranged from −30.4 kcal/mol to −208.3 kcal/mol (Table S4). Among them, 36 (20.6%) and 55 (31.3%) miRNA candidates had more than 1,000 reads in the T48 and CK libraries, respectively. Most of these novel miRNAs were more highly expressed than the known miRNAs. Information about the miRNA candidates identified from the two libraries is summarized in Table S4 and the hairpin structures for the precursors of eight novel miRNAs were chosen as examples and are shown in Fig. 9.

### Differentially expressed miRNAs after *S*. *sclerotiorum* infection

**Figure 10:**
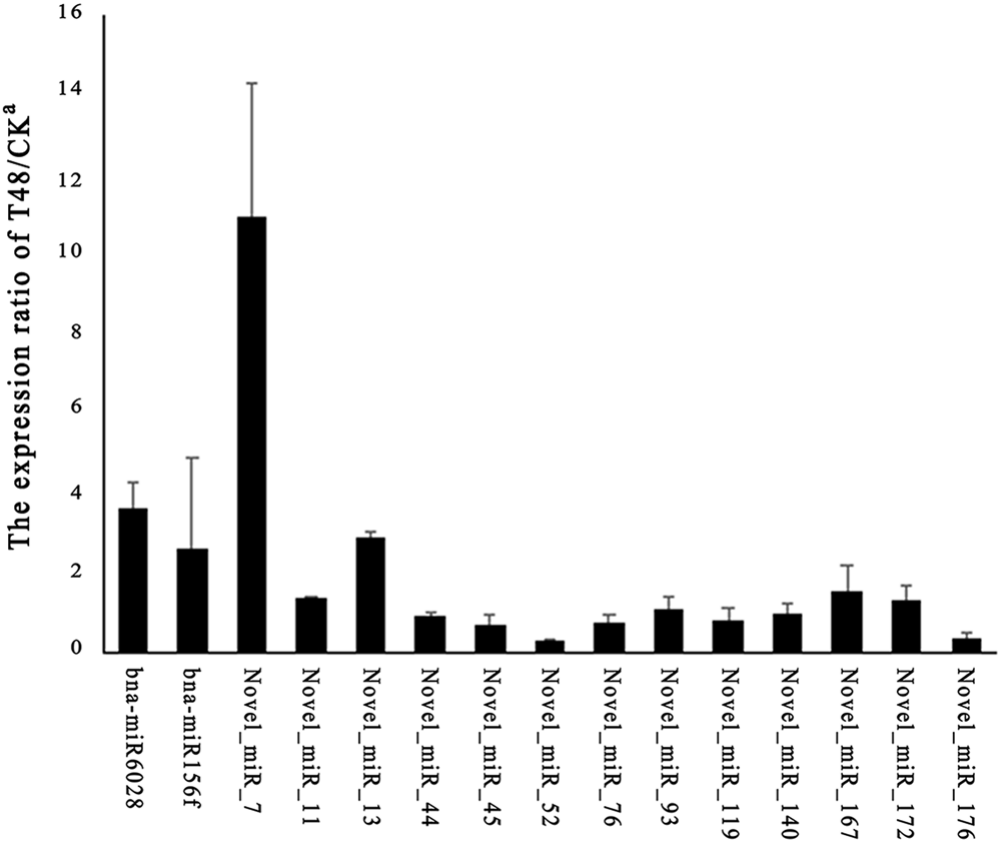
RT-qPCR validation of the miRNAs. The expression values presented are the means of three technical replicates. U6 was used for each sample as an endogenous control. (**A**,**B**) Represent the expression pattern of mature conserved and novel miRNAs, respectively.

To screen for *S*. *sclerotiorum-*responsive miRNAs, differential expression analysis of the miRNAs was performed between the T48 and CK libraries, based on the normalized read count for each identified miRNA. In our study, 10 known and 41 novel miRNAs were identified as differentially expressed (DE) miRNAs based on the following criteria: fold change (≥−2 or ≤2) and FDR-value (≤0.001). Among them, only one known (bna-miR6028) and six novel (Novel_miR_34, Novel_miR_46, Novel_miR_49, Novel_miR_74, Novel_miR_129, and Novel_miR_167) DE miRNAs were down-regulated after *S*. *sclerotiorum* infection. To confirm the reliability of the data produced through deep sequencing, qRT-PCR was conducted. Fifteen miRNAs including two conserved miRNAs (miR6028 and miR156f) and 13 novel miRNAs (Novel_miR_7, Novel_miR_11, Novel_miR_13, Novel_miR_44, Novel_miR_45, Novel_miR_52, Novel_miR_76, Novel_miR_93, Novel_miR_119, Novel_miR_140, Novel_miR_167, Novel_miR_172, and Novel_miR_176) from miRNAs detected in our study were selected (Fig. 10). As expected, the qRT-PCR results were consistent with the deep sequencing results.

### Target prediction of DE miRNAs

To gain insights into functions of DE miRNAs, corresponding target genes were predicted using bioinformatics analysis. Among these target genes, only 15 genes were DEG in our RNA-Seq analysis (Table 4). Of them, eight targets (encoding squamosa promoter-like proteins and phototropic-responsive NPH3 family protein) were targeted by miR156, four genes (encoding revolute and homeobox gene 8 proteins) were targeted by miR166f and the rest three were novel miRNAs (Novel_miR_87/126/158). Interestingly, all 15 targets were reverse expression patterns with corresponding miRNAs but *BnaA08g11090D*, which was encoding CAN OF WORMS1 (COW1).

**Table 4:** DE miRNAs with corresponding DE genes in our study.

DE-miRNAs	log2FC^a^	Targets	log2FC^b^	AGI accession	Functions
bna-miR156a-g	1.05–1.25	BnaA04g27550D	−2.54	AT3G57920	squamosa promoter binding protein-like 15 (SPL15)
bna-miR156a-g	1.05–1.25	BnaA06g36780D	−2.15	AT5G43270	squamosa promoter binding protein-like 2 (SPL2)
bna-miR156a-g	1.05–1.25	BnaA09g27960D	−2.45	AT1G27360	squamosa promoter-like 11 (SPL11)
bna-miR156a-g	1.05–1.25	BnaC04g02520D	−4.13	AT2G42200	squamosa promoter binding protein-like 9 (SPL9)
bna-miR156a-g	1.05–1.25	BnaC07g17030D	−1.83	AT5G43270	squamosa promoter binding protein-like 2 (SPL2)
bna-miR156d/e/f	1.16	BnaAnng03450D	−2.59	AT3G44820	Phototropic-responsive NPH3 family protein
bna-miR156d/e/f	1.16	BnaC01g23990D	−2.00	AT3G44820	Phototropic-responsive NPH3 family protein
bna-miR156d/e/f	1.16	BnaC06g32630D	−3.83	AT1G71690	Protein of unknown function (DUF579)
bna-miR166f	1.09	BnaA02g06170D	−2.50	AT5G60690	REVOLUTA (REV)
bna-miR166f	1.09	BnaA08g11980D	−4.24	AT4G32880	homeobox gene 8 (HB-8)
bna-miR166f	1.09	BnaA10g13520D	−2.06	AT5G60690	REVOLUTA (REV)
bna-miR166f	1.09	BnaAnng30670D	−3.68	AT4G32880	homeobox gene 8 (HB-8)
Novel_miR_87	1.02	BnaC03g36760D	−2.13	AT3G10740	alpha-L-arabinofuranosidase 1 (ASD1)
Novel_miR_129	−1.72	BnaA08g11090D	−2.30	AT4G34580	CAN OF WORMS1 (COW1)
Novel_miR_158	3.32	BnaC02g06060D	−5.89	AT5G15450	casein lytic proteinase B3 (CLPB3)

Note: a and b: log2FC values of miRNAs and genes, respectively.

### Degradome analyses

**Figure 11:**
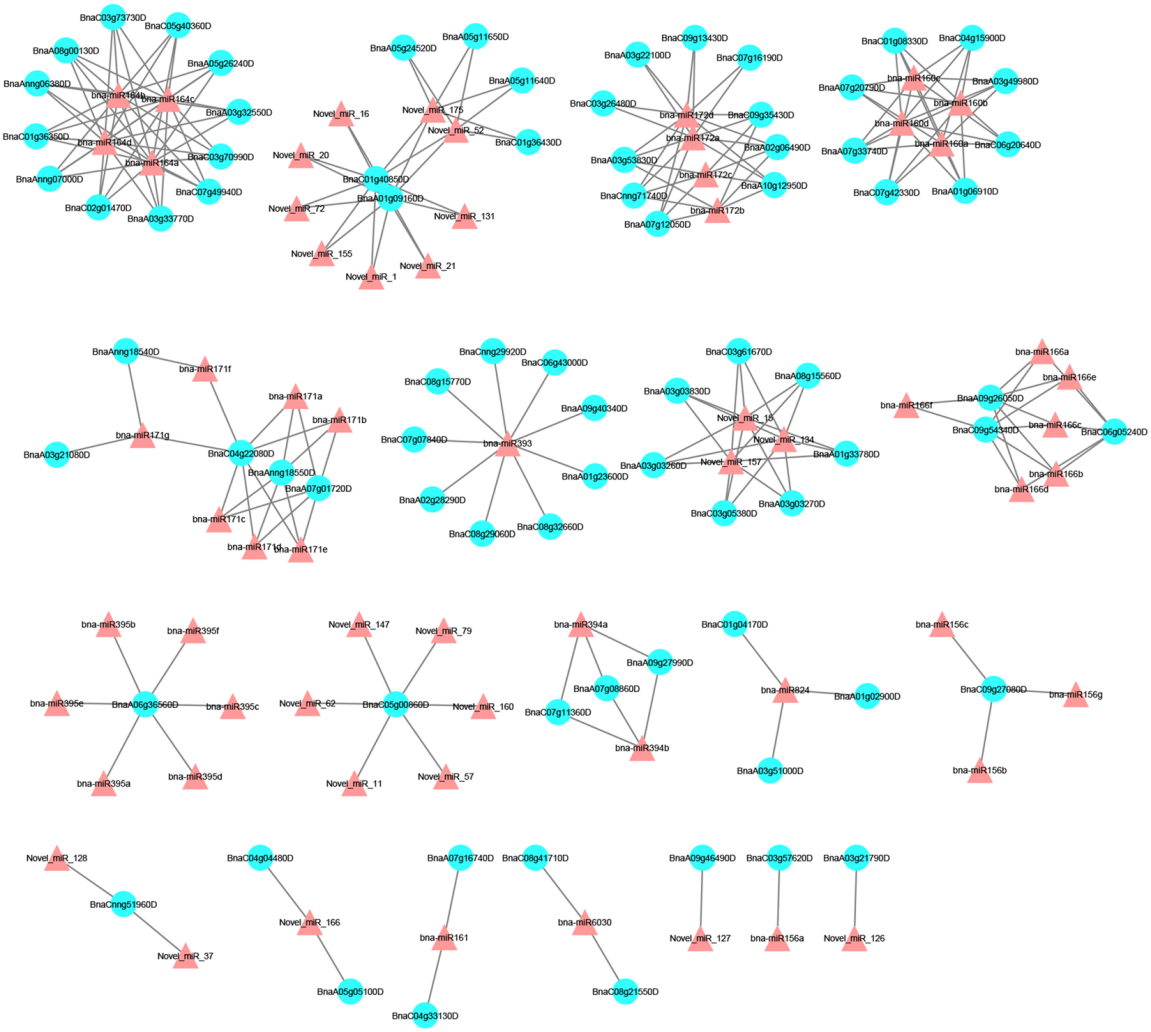
A miRNA-mediated *S*. *sclerotiorum*-responsive regulatory network. Triangles represent the differentially expressed miRNAs and Blue circles represent the target genes of differentially expressed miRNAs.

**Figure 12:**
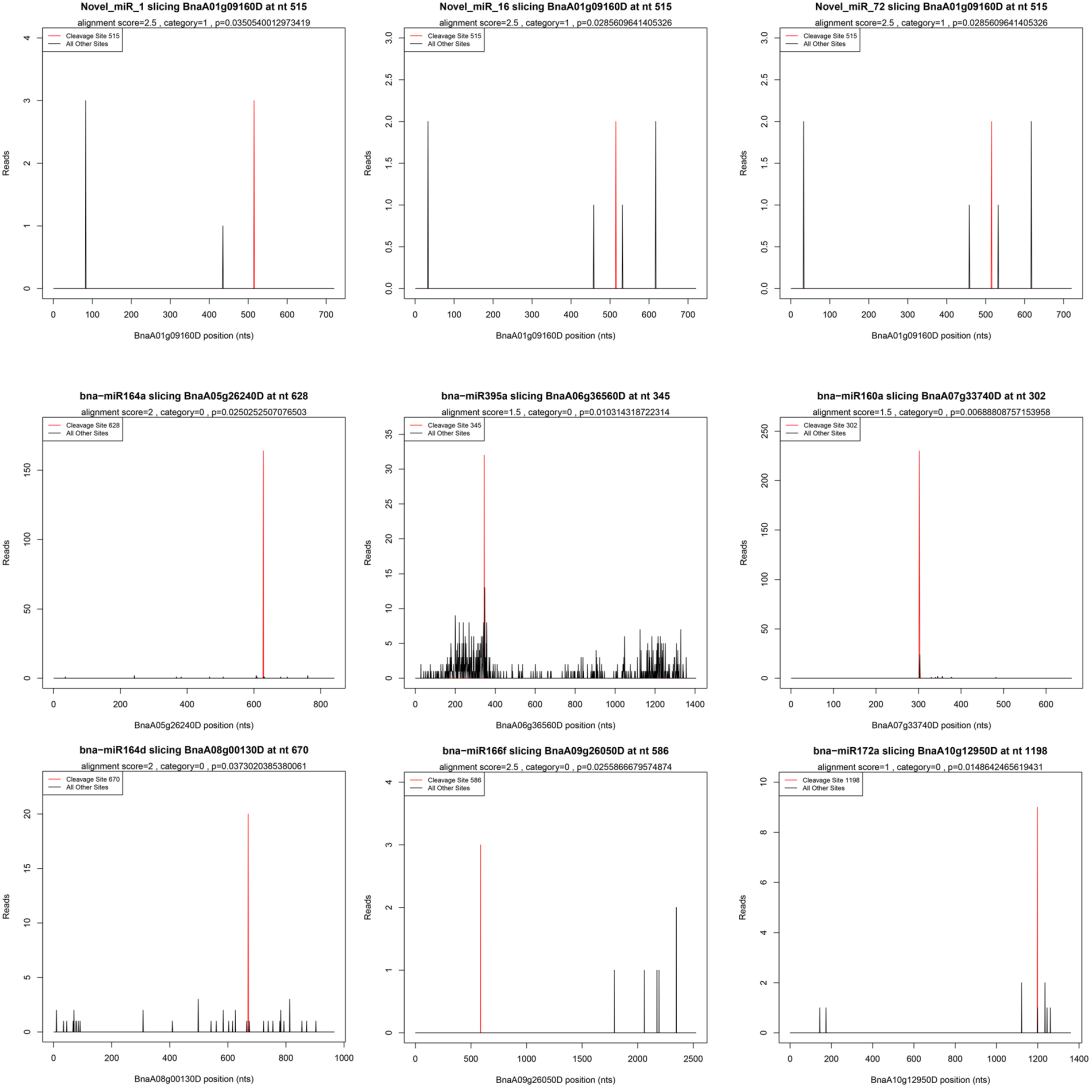
Target plots (t-plots) of miRNAs targets confirmed by degradome sequencing. In t-plots, the red lines indicate the miRNA-directed cleaved transcript. The X axis indicates the nucleotide position in target cDNA. The Y axis indicates the number of reads of cleaved transcripts detected in the degradome cDNA library. “alignment score” is the score for mismatch. Score = 0 represents perfect match and G:U = 0.5.

To identify the potential functions of the miRNAs found in our study, two degradome libraries were constructed to identify the targets of the miRNAs. After sequencing, a total of 13,835,867 and 12,764,316 raw reads with 12,936,736 (93.50%) and 11,910,513 (93.31%) clean reads were obtained in the T48 and CK libraries, respectively. The clean tags were mapped to the *B*. *napus* reference genome database and Rfam database using SOAP2.20 software^42^. Using the Cleave-Land v3.0.1 pipeline^43^, a total of 80 (49 in the T48 and 57 in the CK libraries) cleavage sites with 64 miRNAs were predicted with a *P*-value < 0.05 (Table S5). Most of the targets of the known miRNAs were TFs (Table S5). For example, SBP (bna-miR156), ARF (bna-miR160), NAC (bna-miR164), TOE (bna-miR172), bHLH (bna-miR393), AGL16 (bna-miR824), AFB2/3 (bna-miR393), GRAS (bna-miR171), TIR1 (bna-miR393), and RAP (bna-miR172) were detected in our study (Table S5). Many TFs were also detected in the targets of the novel miRNAs, including DCL1 (Novel_miR_11/57/62/79/147/160), TCP (Novel_miR_1/16/20/21/52/72/131/155/175), and MYB (Novel_miR_37/126/128) (Table S5). Additionally, eight disease resistance protein (TIR-NBS-LRR class) family genes were cleaved by three novel miRNAs (Novel_miR_15/134/157). The interactions between the miRNAs and their targets were presented using cytoscape software (Fig. 11). Nine random miRNA:mRNA pairs were chosen as examples and corresponding t-plots were constructed (Fig. 12).

## Discussion

Due to a lack of resistant germplasm, Sclerotinia stem rot, caused by the pathogen *S*. *sclerotiorum*, results in yield and economic losses in oilseed rape worldwide each year. A better understanding of the molecular mechanisms of resistance to the *S*. *sclerotiorum* pathogen is imperative for long-term, sustainable production of oilseed rape. In our previous study, five relatively resistant *B*. *napus* lines were identified^10^. In this study, we conducted deep sequencing at the transcriptional and post-transcriptional levels to explore the defense response to *S*. *sclerotiorum* infection in the relatively resistant lines. Our data showed that a coordination among different pathways is involved in the resistance response in oilseed rape at the transcriptional and post-transcriptional levels and further analysis is needed regarding candidate genes, miRNAs, or targets identified in our study.

Previous studies on the transcriptomic changes in oilseed rape in response to *S*. *sclerotiorum* infection were primarily conducted using oligonucleotide microarrays. More than 300 DEGs involved in JA biosynthesis and signaling, reactive oxygen species metabolism, and cell wall structure and function were identified using microarrays^44^. Later, 686 and 1,547 DEGs in resistant and susceptible *B*. *napus* genotypes, respectively, associated with *S*. *sclerotiorum* infection using an *Arabidopsis* microarray were detected^39^. Changes in the expression of PR proteins, kinases, transporters, JA, ET, cell wall proteins, and unknown proteins were identified. Researchers further investigated altered gene expression in ZhongYou 821 (ZY821, a partially resistant line) and Westar (a susceptible cultivar) after *S*. *sclerotiorum* infection using a *B*. *napus* microarray^17^. Differential gene expression was identified in genes encoding defense proteins such as osmotins, chitinases, lectins, glucanases, JA, ET, auxin, zinc fingers, WRKYs, AP2, and MYB TFs. Genes involved in molecular processes, such as carbohydrate and energy metabolism, and glucosinolate and phenylpropanoid biosynthesis were also discussed. In our study, genes encoding protein kinases, signal transduction proteins (CDPKs, G proteins, and MAPKs), TFs (WRKYs and MYBs), hormones (JA and ET), PR proteins, secondary metabolism, transporters, P450, UGTs, and lipid metabolism were found to be differentially expressed between the *S*. *sclerotiorum* inoculated and mock-inoculated samples (Tables S6–S13). In other plant-pathogen interactions, such as flax-*Fusarium oxysporum*^45^, banana-*F*. *oxysporum*^46^ and cabbage-*F*. *oxysporum*^47^, the defense proteins or pathways discussed above were also changed.

### Protein kinase genes involved in the recognition of *S*. *sclerotiorum*

The receptor-like kinase (RLK) family plays important roles in the *B*. *napus*-*S*. *sclerotiorum* interaction. More than 1,200 RLK homologs genes in *B*. *napus* were identified using 300 *AtRLKs* (http://www.arabidopsis.org/browse/genefamily/Receptor_kinase. jsp) as queries^24^. In our study, the expression of 214 *BnRLK* genes was altered after inoculation with *S*. *sclerotiorum*, with 158 genes being down-regulated and 56 genes being up-regulated (Table S6). Nucleotide binding site leucine-rich repeat (NBS-LRR) genes, characterized as R genes, have been found to be involved in plant-pathogen interactions^48^. In our study, the expression of 29 NBS-LRR genes was altered after inoculation with *S*. *sclerotiorum*, with 21 genes being down-regulated and 8 genes being up-regulated (Table S6).

### Signal transduction is involved in *B*. *napus*-*S*. *sclerotiorum* interactions

Mitogen-activated protein kinase (MAPK) cascades, which consist of the MAPKKK-MAPKK-MAPK module, have been characterized in the response to pathogen infection^49^. In the current study, 28 *MAPKKK*, one *MAPKK*, and five MAPK genes were induced at 48 hours after inoculation with *S*. *sclerotiorum*. Among these, 21 *MAPKKK* and five *MAPK* genes were down-regulated, and one *MAPKK* (*BnaC02g22230D*) gene was up-regulated by more than 16-fold at 48 hpi with *S*. *sclerotiorum* (Table S7). G-proteins transduce detection signals from RLKs^50^. In our study, seven G-protein transcripts were up-regulated at 48 hpi with *S*. *sclerotiorum* (Table S7). Calcium (Ca^2+^), acting as an essential second messenger, is involved in the plant’s response to abiotic and biotic stresses^51^. In our study, 91 calcium-related genes, such as calcium-binding EF-hand family proteins, calcium-dependent protein kinase (CDPK), and calmodulin binding protein (CAM), showed differential expression (most of them were down-regulated) (Table S7). In another gene family, *MILDEW RESISTANCE LOCUS O* (*MLO*), *MLO2*, *MLO8*, and *MLO12* were down regulated, while three copies of *MLO6* were up regulated in the *S*. *sclerotiorum-*inoculated samples. (Table S7).

### Transcriptional regulation

TFs play crucial roles in response to pathogen attack^52^, and 602 TFs belonging to 39 families were identified in this study (Fig. 1E). In total, 51 *WRKY* genes were differently expressed after *S*. *sclerotiorum* infection. Among these, 18 genes (one *WRKY3*, three *WRKY6s*, one *WRKY 14*, two *WRKY28s*, two *WRKY29s*, two *WRKY42s*, one *WRKY57*, one *WRKY61*, one *WRKY72*, and four *WRKY75s*) were up-regulated (Table S8). MYB TFs, which comprise a large TF family, have central roles in pathogen responses^53^. In this study, 67 MYB TF transcripts were differentially expressed at 48 hpi. Of them, 25 MYBs genes were up-regulated in the *S*. *sclerotiorum*-inoculated plants (Table S8).

### Hormone regulation

Besides their functions in plant growth and development, plant hormone-regulated genes have important roles in plant biotic stress response^54^. The biosynthesis or signal transduction of hormones, such as CK, IAA, BR, SA, JA, ET, ABA, and GA, were affected at 48 hpi with *S*. *sclerotiorum* (Fig. 1F). As a positive regulator, JA plays important roles in plant disease resistance^55^. In this study, several transcripts encoding proteins involved in JA biosynthesis, including jasmonic acid carboxyl methyltransferase (*JMT*), allene oxide synthase (*AOS*), allene oxide cyclase 2 (*AOC2*), fatty acid desaturase (*FAD*), and lipoxygenase (*LOX*) family proteins, were differently expressed in the *S*. *sclerotiorum* infected samples (Table S9). Five transcripts encoding jasmonate-zim-domain protein 3 (JAZ3), which negatively regulates JA transcriptional activity, were down-regulated at 48 hpi with *S*. *sclerotiorum* (Table S9). ET, which is involved in the regulation of growth and development, acts synergistically with JA in responses to environmental stresses^54^. In our study, key enzymes involved in ET biosynthesis, including 1-aminocyclopropane-1-carboxylatesynthase (*ACS*) and 1-aminocyclopropane-1-carboxylateoxidase (*ACO*), were up-regulated in the *S*. *sclerotiorum*-inoculated samples, except for one copy (*BnaC02g15560D*) of ACS12. Moreover, ET signaling pathway genes, including *ethylene response sensor* (*ERS*), *ethylene response factor* (*ERF*), and *octadecanoid-responsive Arabidopsis AP2/ERF 59* (*ORA59*), and their regulated genes, such as *pathogenesis-related 4 (PR4)* and *plant defensin 1*.*2* (*PDF1*.*2*), were detected in our study (Table S9).

### PR proteins

Pathogenesis-related genes (*PRs*) play central roles in disease resistance^56^. In our study, the expression of 19 genes encoding pathogenesis-related 4 (PR4), pathogenesis-related family protein, pathogenesis-related gene 1 (PR1), and pathogenesis-related thaumatin superfamily proteins, was altered. Of them, eight genes encoding pathogenesis-related thaumatin superfamily proteins were down-regulated and the rest were up-regulated in the *S*. *sclerotiorum*-inoculated samples (Table S10). Chitinase degrades chitin, which is the primary structural component of fungal cell walls^57^. Seventeen genes encoding chitinase proteins were induced after *S*. *sclerotiorum* infection (Table S10). Among these, two genes (*BnaC07g30330D* and *BnaA06g26630D*) encoding chitinase A (CHIA) and one gene (*BnaA05g03440D*) encoding a chitinase family protein were down-regulated at 48 hpi, while the other 14 genes encoding chitinase family proteins were up-regulated.

### Secondary metabolism

Secondary metabolites play crucial roles in plant defense against pathogen attack^58^. Genes involved in the biosynthesis of phenylpropanoids, flavonoids, isoprenoids, glucosinolates, and wax were differentially expressed after *S*. *sclerotiorum* infection in our study.

Phenylpropanoid is one of the most important secondary metabolites involved in stress-defense^59^. Fourty-three genes encoding components of phenylpropanoid metabolism were detected and 12 of them were up-regulated at 48 hpi with *S*. *sclerotiorum*. In addition, 21 genes involved in lignin formation (including cinnamicacid 3-hydroxylase (*C3H*), cinnamicacid 4-hydroxylase (*C4H*), cynnamoyl-CoA reductase (*4CL*), cinnamyl alcohol dehydrogenase (*CAD*), caffeoyl-CoA O-Methyltransferase (*CCoAOMT*), cinnamoyl-CoA reductase1 (*CCR1*), caffeic acid O-methyltransferase (*COMT*), ferulate 5-hydroxylase (*F5H*), and shikimate quinatehydroxy cinnamoyl transferase (*HCT*)) were differently expressed in our study (Table S11).

Owning to their high antioxidant capacity, flavonoids have been used in transgenic engineering to increase resistance to pathogen attack^60^. Fourty-two key genes encoding proteins related to flavonoid biosynthesis, including flavonol synthase, chalcone synthase, flavonoid 3″-monooxygenase, chalcone isomerase, anthocyanin 5-aromatic acyltransferase, and dihydro flavonol reductase (DFR), were differentially expressed in the *S*. *sclerotiorum-*infected samples. Ten out of 14 genes involved in anthocyanin biosynthesis were up-regulated in the *S*. *sclerotiorum-*infected samples, and two genes, BnaA04g03320D and BnaC04g25200D, were *jasmonate-regulated gene 21* (*JRG21*) genes with a 5.65 and 9.35 log2-fold increase, respectively. Interestingly, all seven chalcones synthesis genes and all eight dihydroflavonols genes, including *TRANSPARENT TESTA*, *DFR*, and *F3H*, were down-regulated at 48 h after *S*. *sclerotiorum* infection. Furthermore, six out of 13 genes associated with flavonol biosynthesis were increased in the *S*. *sclerotiorum*-inoculated plants (Table S11).

Isoprenoids, which are involved in plant growth and development, have specialized functions in response to pathogen attack^61^. Thirty-five genes related to the metabolism of carotenoids, terpenoids, and tocopherol biosynthesis were differentially expressed at 48 hpi with *S*. *sclerotiorum*. Most of them, including *terpene synthase 21* (*TPS21*), *LUTEIN DEFICIENT 2* (*LUT2*), and *zeta-carotene desaturase* (*ZDS*), were down-regulated in the *S*. *sclerotiorum*-inoculated plants. Three copies of *phytoene desaturation 1* (*PDS1*), involved in tocopherol biosynthesis, had a 3.05 to 4.32 log2-fold increase (Table S11).

Genes related to glucosinolate synthesis, another secondary metabolite, were found to be differentially expressed in our study. Two genes involved in glucosinolate synthesis were induced while 19 genes were suppressed at 48 hpi with *S*. *sclerotiorum* (Table S11). The two up-regulated genes, *BnaC03g30410D* and *BnaCnng43090D*, encoding AOP1 and SUR1, respectively, are responsible for the synthesis of indole glucosinolates^62^.

### Transport

Altered expression was observed in genes involved in transport pathways, such as ABC transporters, major intrinsic proteins (MIPs), and amino acid transporters/permeases at 48 hpi with *S*. *sclerotiorum*.

Multidrug transporters, comprised of MATE efflux proteins and ABC transporters, remove noxious compounds from cells^63^. Fifty-five multidrug transporter genes were found to have altered expression in this study, including 26 with increased abundance after *S*. *sclerotiorum* infection. Of the multidrug transporter genes, seven MATE efflux family proteins, nine multidrug resistance-associated proteins (MRPs), eight pleiotropic drug resistance proteins (PDR), nine ATP-binding cassette proteins (ABC), two ABC transporter family proteins, and 11 ABC-2-type transporter family proteins aid in secondary metabolite transport to resist pathogens (Table S12).

MIPs, including NOD26-like intrinsic protein (NIPs), plasma membrane intrinsic proteins (PIPs), and tonoplast intrinsic proteins (TIPs), play crucial roles in water and solute transport and plant stress responses^64^. Seven NIPs, 16 PIPs, and seven TIPs were differently expressed at 48 hpi with *S*. *sclerotiorum* (Table S12), with most of these genes being down-regulated. These expression changes may be regulated by the pathogen to improve invasion into plant tissues^65^; alternatively, these membrane proteins could promote the conduction of hydrogen peroxide (H_2_O_2_) to support defense signaling^66^.

In our study, 28 differentially expressed amino acid transporter genes were detected, including amino acid permeases (AAPs), bidirectional amino acid transporter 1 (BAT1), cationic amino acid transporter (CAT), and transmembrane amino acid transporter family proteins (Table S12). Two transcripts encoding AAP3 (*BnaC06g38080D* and *BnaA07g33510D*) were up-regulated at 48 hpi with *S*. *sclerotiorum*.

### Other genes

Differential expression of numerous other gene groups involved in the response to pathogen attack were identified in our study. Many cytochrome family protein (CYP) P450 genes, a large group of genes involved in diverse metabolic processes, were up-regulated (about 50%) at 48 hpi with *S*. *sclerotiorum* (Table S13). Many UDP-glucosyl transferase family proteins (UGTs), which transfer UDP-glucose to diverse secondary metabolites or hormones, were up-regulated at 48 hpi with *S*. *sclerotiorum*. One transcript, *BnaA01g03020D* encoding UGT73B3, which is involved in abscisic acid glucosyltransferase, was the most up-regulated of the UGTs with a 6.59 log 2-fold change (Table S13). Groups of genes involved in modifying or degrading other proteins were found to be differently expressed. These transcripts encode enzymes including 11 aspartate proteases, 20 cysteine proteases, 16 serine proteases, 14 subtilases, and 168 ubiquitin genes. Most of these genes were down-regulated in the *S*. *sclerotiorum*-infected samples (Table S13). Finally, DEGs encoding enzymes involved in lipid metabolism were also observed. For example, seven transcripts encoding alpha beta hydrolases were up-regulated while all five *SHAVEN 3* (*SHV3*) genes were down-regulated at 48 hpi with *S*. *sclerotiorum* (Table S13).

As post-transcriptional gene regulators, miRNAs are involved in plant development and various stress responses, including plant–fungus interactions, by suppressing corresponding target genes^67^. Using next generation sequencing technologies, several fungi-responsive miRNAs were identified in *A*. *thaliana*^68^, Paulownia^69^, wheat^70^, and oilseed rape^41^. However, only several studies to date have reported using deep sequencing approaches to identify miRNAs and their expression in *B*. *napus* in response to *S*. *sclerotiorum* infection. What noteworthy is that 280 *B*. *napus* miRNA candidates small RNAs from both normal and *S*. *sclerotiorum* inoculated leaves were identified using high-throughput deep sequencing technologies and over-expression of *AGO1* and *AGO2* could decrease resistance to *S*. *sclerotiorum* in oilseed rape^71^. In the current study, sRNA-seq approach was used to identify the miRNAs responsive to *S*. *sclerotiorum* infection and degradome sequencing was performed to identify the targets of corresponding miRNAs. In our study, 77 known and 176 novel miRNAs were detected (Tables 2, S4), and 10 known and 41 novel miRNAs were considered as differentially expressed miRNAs after *S*. *sclerotiorum* infection (Fig. 8B,C).

In plants, miRNAs play key roles in many biological processes and in the response to various stresses by regulating their corresponding target genes^29^. Many of the target genes of miRNAs are TFs^72^. In our degradome sequencing data, miRNA393, an important miRNA which is involved in many processes, cleaved seven genes encoding auxin signaling F-box 2/3 (AFB2/3), TRANSPORT INHIBITOR RESPONSE 1 (TIR1), and basic helix-loop-helix (bHLH) DNA-binding superfamily proteins with diverse functions. A previous study on the interactions between auxin signaling and miR393 showed that over-expression of miR393 in *A*. *thaliana* lines resulted in greater susceptibility to necrotrophic pathogens, and that auxin signaling repressed SA levels and signaling^29^. Furthermore, higher glucosinolate contents and lower camalexin levels were also detected in the miR393 over-expression lines^31^. In *B*. *napus*–*V*. *longisporum* interactions, modulation of the miR168-Argonaute 1 (AGO1) interaction played key regulatory roles^41^. Unfortunately, miR168 was not differentially expressed in our study, and its target, *AGO1*, was not detected. bna-miR6028, which targets receptor like protein 27 (RLP27) and jasmonate-zim-domain protein 10, which are responsive to stress and wounding^73^, was significantly down-regulated at 48 hpi with *S*. *sclerotiorum*. The target genes of bna-miR164 and bna-miR395, which encode NAC proteins and AOC2, respectively, were responsive to oxidative stress^74^, cold^75^, and salt stress^76^. Taken together, the results illustrated that cross-talk exists between miRNAs and target gene pathways, thereby regulating the response to fungal pathogens and other stresses in *B*. *napus*.

## Materials and Methods

### Fungal and plant materials

A strain of *S*. *sclerotiorum* 1980 was provided by Dr. Jiaqin Mei, Chongqing Engineering Research Center for Rapeseed, College of Agronomy and Biotechnology, Southwest University, and was cultured on potato dextrose agar media (PDA, Becton Dickinson, Sparks, MD, USA) at 22 °C in darkness. Five relatively resistant *B*. *napus* lines used in this experiment were identified and selected in our previous study^10^. Three plants per line and two sites per plant were used to detect the response to *S*. *sclerotiorum* infection and bulked for mRNA, sRNA and degradome analysis. Pathogen inoculation and tissue harvest were conducted following the procedures described in our previous study^10^. For verifying its accuracy, ZS11 was used to detect genes response to *S*. *sclerotiorum* infection. Stem tissues from inoculated (48 hpi) and control plants were obtained, frozen immediately in liquid nitrogen, and stored at −80 °C.

### RNA isolation and high-throughput sequencing

Total RNA was extracted from stems of each treatment (inoculated and mock-inoculated controls) using TRIZOL reagent (TianGen, China) according to the manufacturer’s instructions. At least five plants per treatment and two sites per plant (10 sites) were mixed for each sample. DNA contamination was removed from the total RNA using RNA-free DNase I (Promega). A Qubit Fluorometer was used to determine the RNA quantity and quantity and an Agilent 2100 Bioanalyzer was used to detect the RNA purity and integrity. Two RNA samples were sent to Beijing Biomarker Technologies Co., Ltd. (Beijing, China) for mRNA, sRNA, and degradome sequencing using an Illumina HiSeqTM 2500. The original mRNA, sRNA, and degradome sequencing data were deposited in the NCBI Sequence Read Archive with accession nos. SRP096626, SRP096847, and SRP0596850, respectively.

### mRNA sequencing data analysis

After removal of the adapter sequences and low quality sequences, clean reads were mapped to the *B*. *napus* reference genome (http://www.genoscope.cns.fr/brassicanapus/) and were then assembled using TopHat 2.0.0 and DEGseq. Gene expression levels were estimated using the FPKM (fragments per kilobase of exon per million mapped fragments) values and DEGs were identified with Cuffdiff using the following two criteria: (i) false discovery rate (FDR) *p*-value correction of <0.05 and (ii) |log2 (fold change)| >1.

To screen for possible TF and plant hormone genes, the DEGs were aligned to known TF and plant hormone genes, which were downloaded from The Plant Transcription Factor Database (http://planttfdb.cbi.pku.edu.cn/index.php) and the *Arabidopsis* Hormone Database 2.0 (http://ahd.cbi.pku.edu.cn/), respectively.

### Identification of known and novel miRNAs in *B. napus*

The clean reads were obtained after removing low-quality reads, contaminants, and adaptors using the SOAPnuke software (http://soap.genomics.org.cn/)^42^. The length distribution of the clean reads was categorized and then the clean reads were immediately used to search GenBank and the Rfam 11.0 database (http://rfam.sanger.ac.uk/) to annotate the noncoding RNAs. Subsequently, the sRNAs were aligned to miRNA precursors of rapeseed in miRBase 21.0 to obtain known miRNAs based on the criteria of our previous publication^25^. The remaining unannotated reads were used to predict novel miRNAs using criteria described before^77^.

### Analysis of differential expression conserved and novel miRNAs and target prediction

The expression of each miRNA was normalized using the formula: normalized expression = actual miRNA count/total count of clean reads (×1,000,000). The fold change of each miRNA after inoculation was calculated as (fold change = log2 (miRNA_T48_/miRNA_CK_)). miRNAs with fold changes ≥1 or ≤−1 and with *P* ≤ 0.01 were regarded as up-regulated or down-regulated, respectively. The TargetFinder software was used for target prediction of significantly differentially expressed miRNAs in response to *S*. *sclerotiorum* infection using the previously described scoring system and criteria^25^.

### Degradome sequencing data analysis

For degradome sequencing, two library constructions were performed on an Illumina HiSeq2500 sequencing system according to the manufacturer’s instructions. Clean reads were filtered from the raw data and sRNAs were aligned to GenBank and Rfam 11.0 databases for annotation of the cleaved target genes. Furthermore, CleaveLand v3.0.1 (August 26, 2011)^78^ was used to predict the miRNA cleavage sites and T-plot figures were drawn.

### Functional annotation of DEGs and target genes

For annotation of gene function, GOatools (https://github.com/tanghaibao/GOatools) was used to analyze Gene Ontology (GO) term enrichment for the DEGs and the predicted targets identified above, and enriched terms, in which the FDR was less than 0.01, were displayed using the online tool WEGO (Web Gene Ontology annotations Plot, http://wego.genomics.org.cn). The KOBAS2.0 website (http://kobas.cbi.pku.edu.cn/home.do) was used to analyze the enrichment of the DEGs in the KEGG (Kyoto Encyclopedia of Genes and Genomes) pathway and MapMan software was used to analyze the metabolic pathways and functional classifications of the DEGs.

### Validation of sequencing data by qRT-PCR

To validate the reliability of the sequencing data, 55 and 15 randomly selected DEGs and significantly differentially expressed miRNAs, respectively, were selected for qRT-PCR analysis. Total RNA was used for library construction and the qRT-PCR procedures for the DEGs and miRNAs were described in previous papers^10,25^. Actin7 and U6 snRNA of *B*. *napus* were used as the references for genes and miRNAs, respectively. All primers used for qRT-PCR are listed in Table S1. Three technical replicates were performed for each reaction.

## Electronic supplementary material

S1-S13 Supporting information
